# Effects of Loneliness and Social Isolation on Cognitive Health: Latest Perspectives and Future Directions

**DOI:** 10.1093/geroni/igab046.916

**Published:** 2021-12-17

**Authors:** Kheng Siang (Ted) Ng, Kexin Yu, James Lubben

## Abstract

Loneliness and social isolation as antecedents of cognitive decline have received substantial attention in recent research. This symposium addresses this year’s conference theme of aging in the “new normal”. The COVID-19 pandemic has highlighted the negative impacts of loneliness and social isolation on older adults’ health and wellbeing. This symposium includes studies that shed light on the relationships between loneliness, social isolation and cognitive health using a multidisciplinary approach, and provide recommendations and future directions for advancing this research area. The first presentation examines cardiovascular biomarkers as potential mechanisms that mediate the longitudinal relationship between loneliness and cognitive decline with the HRS dataset. The second presentation examines several social isolation indicators and their effects on cognitive decline in a Canadian longitudinal study. Using the US ADRC longitudinal study of aging, the third study shows the effect of loneliness on cognitive health in older adults pre- and post-onset of the COVID-19 pandemic. The symposium concludes with a literature review of the different measures employed to operationalize the constructs of loneliness, isolation, which resulted in heterogeneous study findings on their influences on the risk of developing dementia. This review calls for consistent measures to produce comparable evidence on the health consequences of loneliness and isolation. In all, this symposium reports and reviews the latest evidence on the association between social isolation, loneliness and cognitive health amidst the ongoing COVID-19 pandemic. It also echoes the conference theme of transforming disruption to opportunities in aging health service and research.

